# A transferrable IncL/M plasmid harboring a gene encoding IMP-1 metallo-β-lactamase in clinical isolates of *Enterobacteriaceae*

**DOI:** 10.1186/s12879-021-06758-5

**Published:** 2021-10-13

**Authors:** Nobuyoshi Mori, Tatsuya Tada, Satoshi Oshiro, Kyoko Kuwahara-Arai, Teruo Kirikae, Yuki Uehara

**Affiliations:** 1 Department of Infectious Diseases, St. Luke’s International Hospital, Tokyo, Japan; 2 Department of Microbiology, Juntendo University Graduate School of Medicine, 2-1-1 Hongo, Bunkyo-ku, Tokyo, 113-8421 Japan; 3 Department of Clinical Laboratory, St. Luke’s International Hospital, Tokyo, Japan

**Keywords:** Carbapenemase producing *Enterobacteriaceae*, IncL/M, IMP-1 metallo-β-lactamase

## Abstract

**Background:**

The worldwide spread of carbapenemase-producing *Enterobacteriaceae* (CPE) has reduced the clinical utility of carbapenems. Plasmids often play an important role in the spread of genes encoding drug-resistance factors, especially in the horizontal transfer of these genes among species of *Enterobacteriaceae*. This study describes a patient infected with three species of CPE carrying an identical transferrable IncL/M plasmid.

**Methods:**

Clinical isolates of CPE were collected at St. Luke’s International Hospital, Tokyo, Japan, from 2015 to 2019. Three species of CPE isolates, *Enterobacter cloacae*, *Klebsiella aerogenes* and *Serratia marcescens*, were isolated from a patient who developed severe gallstone pancreatitis associated with bloodstream infection, with all three isolates producing IMP-1 metallo-β-lactamase. The complete sequences of the plasmids of the three isolates were determined by both MiSeq and MinION. The medical chart of this patient was retrospectively reviewed conducted to obtain relevant clinical information.

**Results:**

The three CPE species carried an IncL/M plasmid, pSL264, which was 81,133 bp in size and harbored *bla*_IMP-1_. The genetic environment surrounding *bla*_IMP-1_ consisted of *int1-bla*_IMP-1_*-aac(6’)-IIc-qacL-qacEdelta1-sul1-istB-*IS*21*. Conjugation experiments showed that *S. marcescens* could transmit the plasmid to *E. cloacae* and *K. aerogenes*. In contrast, pSL264 could not transfer from *E. cloacae* or *K. aerogenes* to *S. marcescens*.

**Conclusion:**

The IncL/M plasmid pSL264 harboring *bla*_IMP-1_ was able to transfer among different species of *Enterobacteriaceae* in a patient receiving long-term antimicrobial treatment. The worldwide emergence and spread of IncL/M plasmids harboring carbapenemase-encoding genes among species of *Enterobacteriaceae* is becoming a serious public health hazard.

## Background

Carbapenems are often the treatment of last resort for patients with serious infections caused by gram-negative bacteria. The worldwide emergence of carbapenemase-producing *Enterobacteriaceae* (CPE) has limited the clinical utility of this class of antimicrobial agents [1]. The most common carbapenemases among CPE are *Klebsiella pneumoniae* carbapenemases (Amber class A), IMP-type metallo-β-lactamases (MBLs), VIM-type MBLs, NDM-type MBLs (class B), and OXA-48-like (class D) enzymes [2]. MBLs produced by gram-negative bacteria confer resistance to all β-lactams, except for aztreonam, and are characterized by their efficient hydrolysis of carbapenems [2, 3]. IMP-1 was first discovered in Japan in 1991 [4] and IMP-producing *Pseudomonas aeruginosa* and *Enterobacteriaceae* are frequently detected in patients in the Asia–Pacific region [2, 4–7].

Plasmids often play an important role in the spread of genes encoding drug resistance factors, with these plasmids often involved in the horizontal transfer of drug resistance genes among *Enterobacteriaceae* [8]. For example, the plasmid IncX4, which harbors *mcr-1*, a gene associated with colistin resistance, was shown to easily transfer between *Escherichia coli* and *K. pneumoniae* [9]. In addition, IncX3 plasmids carrying *bla*_NDMs_ are effectively transferred among *Enterobacteriaceae* species and contribute to the worldwide dissemination of NDM producers [10, 11]. IncFII(K) plasmids carrying *bla*_IMPs_ and several Inc type plasmids, including IncL/M, IncN2, IncHI1B-IncFIB (Mar) and IncX3-IncC-like plasmids, carrying *bla*_VIMs_ have contributed to the global spread of *bla*_IMPs_ and *bla*_VIMs_ among different species of *Enterobacteriaceae* [12].

Few reports have described the in vitro and in vivo horizontal transfer of identical carbapenemase-encoding plasmids [13–16]. This study describes a patient infected with three species of *Enterobacteriaceae*, all of which carried a transferrable IncL/M plasmid producing IMP-1.

## Methods

### Bacterial strains and antimicrobial susceptibility

All clinical isolates of *Enterobacteriaceae* not susceptible to carbapenems were prospectively collected and stored at the microbiological laboratory of St. Luke’s International Hospital, Tokyo, Japan, from April 2015 to May 2019. Initial identification and susceptibility tests were performed using Microscan WalkAway 96 Plus (Beckman-Coulter) and Microscan Neg Series NC-EN2J (Beckman-Coulter). Susceptibility results were interpreted in accordance with the Performance Standards for Antimicrobial Susceptibility Testing M100-25 of the Clinical Laboratory Standards Institute (CLSI) [17]. These isolates were screened for production of carbapenemase by a modified carbapenem inactivation method. Thereafter, MBLs were detected using sodium mercaptoacetic acid. During the screening of CPE, one patient was found to harbor three species of CPE, *Enterobacter cloacae*, *Serratia marcescens* and *Klebsiella aerogenes*.

### Identification and whole genome sequencing of CPE harboring *bla*_IMP-1_

The CPE isolates were screened for the presence of *bla*_IMP-1-like_ by PCR using the primers IMP-F (5’-ATGAGCAAGTTATCTGTATTCTTTA-3’) and IMP-R (5’-TTAGTTGCTTGGTTTTGATGGTTTT-3’). All PCR products were sequenced using ABI PRISM 3500XL DNA Analyzer (Applied Biosystems, Foster, CA). The whole genome of each isolate was extracted using DNeasy Blood and Tissue kits (Qiagen, Tokyo, Japan) for MiSeq (Illumina, San Diego, CA) and QIAGEN Genomic-tip 100/G and Genomic DNA Buffer Set (Qiagen) for MinION (Oxford Nanopore Technologies, Oxford, UK). Whole genomes and plasmids of the three isolates were sequenced by MiSeq platform using 600 cycle Reagent Kit v.3 and MinION platform using R9.4 flow cell (FLO-MIN106), according to the manufacturers’ instructions. The sequence reads generated by MiSeq were quality trimmed and filtered using CLC Genomics Workbench v11 (CLC bio, Aarthus, Denmark). MinION data were base called by Guppy v3.6.1 (Oxford Nanopore), trimmed by NanoFilt v2.2 (https://github.com/wdecoster/nanofilt), and adaptors trimmed by Porechop v0.2.3 (https://github.com/rrwick/Porechop). The long reads generated by MinION and the short reads generated by Miseq were assembled and polished using Unicycler v0.4.7 [18]. The sequence of drug resistance genes and plasmid typing were determined using Resfinder 4.1 and PlasmidFinder 2.1, respectively, from the Center for Genomic Epidemiology (CGE) (https://cge.cbs.dtu.dk/services/).

### Structure of the plasmids harboring *bla*_IMP-1_

The sequences of the plasmids in the three isolates were compared using GENETYX-MAC ver. 19.0.1 (GENETYX Co.), and the detailed genetic structure of the plasmid in *E. cloacae* was determined by the Rapid Annotation using Subsystem Technology version 2.0 (https://rast.nmpdr.org) [19]. Whole genome sequences of the plasmids were analyzed by BLAST® website (https://blast.ncbi.nlm.nih.gov/Blast.cgi, accessed September 21, 2020) and in silico MolecularCloning Genomics Edition v7 (in silico biology, Inc. Kanagawa, Japan).

### Conjugation of the plasmid among the isolates

The type strains of *E. cloacae* (NBRC13535), *K. aerogenes* (NBRC13534) and *S. marcescens* (NBRC102204) were plated on Mueller–Hinton broth (MHB) containing 50 µg/ml rifampicin. The grown cells were selected as recipient strains. The donor strains were cultivated in MHB containing ceftazidime and the recipient strains in MHB overnight. The cells were harvested by centrifugation (5000×*g* for 3 min), washed three times with PBS and resuspended in PBS to an OD = 3.5. Donor and recipient cells, mixed at an optimal ratio of 1:3 [20], were incubated for 3 h at 37 ºC and plated on selective media, consisting of MHB containing 50 µg/ml rifampicin or 50 µg/ml ceftazidime and 50 µg/ml rifampicin, for 18 h. The conjugation frequencies of donor cells were calculated based on the number of colonies on each selective medium.

### Multilocus sequence typing and antimicrobial resistance genes

Multilocus sequence typing (MLST) was performed using MLST 2.0 (https://cge.cbs.dtu.dk/services/MLST-2.0/) [21], and antimicrobial resistant genes of *E. cloacae* SL264 were determined using ResFinder 4.1 (https://cge.cbs.dtu.dk/services/ResFinder/) [22].

### Clinical information

The medical chart of the patient was retrospectively reviewed conducted to obtain relevant clinical information.

### Nucleotide sequence accession number

The chromosome and two plasmids (pSL264 and pSL264-2) sequences of *E. cloacae* SL264 have been deposited at GenBank under accession number AP024913, AP024914 and AP024915, respectively. The chromosome and the plasmid (pSL267) sequences of *S. marcescens* SL267 were AP024916 and AP024917, respectively. The chromosome and two plasmids (pSL269 and pSL269-2) sequences of *K. aerogenes* SL269 have been deposited at GenBank under accession number AP024918, AP024919 and AP024920, respectively.

## Results

### Description of the patient

The patient was an 86-year-old man with a history of pancreatic cancer who developed severe gallstone pancreatitis and complicated pancreatic cyst infection. Of note, he was empirically treated with meropenem and a surveillance culture of his pancreatic fluid yielded an IMP-1-producing strain of *E. cloacae,* designated SL264, which was considered colonization (Table 1). Two months after the initial event, an IMP-1-producing strain of *S. marcescens,* designated SL267, was isolated from the pancreatic drainage tube (Table 1). Five days later, however, the patient developed a high fever. Culture of a blood sample resulted in the isolation of an IMP-1-producing strain of *K. aerogenes,* designated SL269 (Table 1). The patient was successfully treated with prolonged infusion of meropenem, colistin and tigecycline for 14 days.

**Table 1:** Characteristics of IMP-1 metallo-β-lactamase producing *Enterobacteriaceae*

Isolates	ST	Tissue source	MIC (μg/mL)						
			AMK	AZT	CAZ	CIP	CST	MPM	TIG
*E. cloacae* SL264	175	Pancreatic cyst	0.5	64	256	1	≤ 0.016	2	1
*S. marcescens* SL267	N/A	Pancreatic cyst	2	≤ 0.25	64	≤ 0.25	> 32	4	≤ 0.25
*K. aerogenes* SL269	N/A	Blood	2	32	256	2	0.063	4	2

ST, sequence type; N/A, not applicable; AMK, amikacin; AZT, aztreonam; CAZ, ceftazidime; CIP, ciprofloxacin; CST, colistin; MPM, meropenem and TIG, tigecycline

### Structure of the plasmid harboring *bla*_IMP-1_

**Fig. 1:**
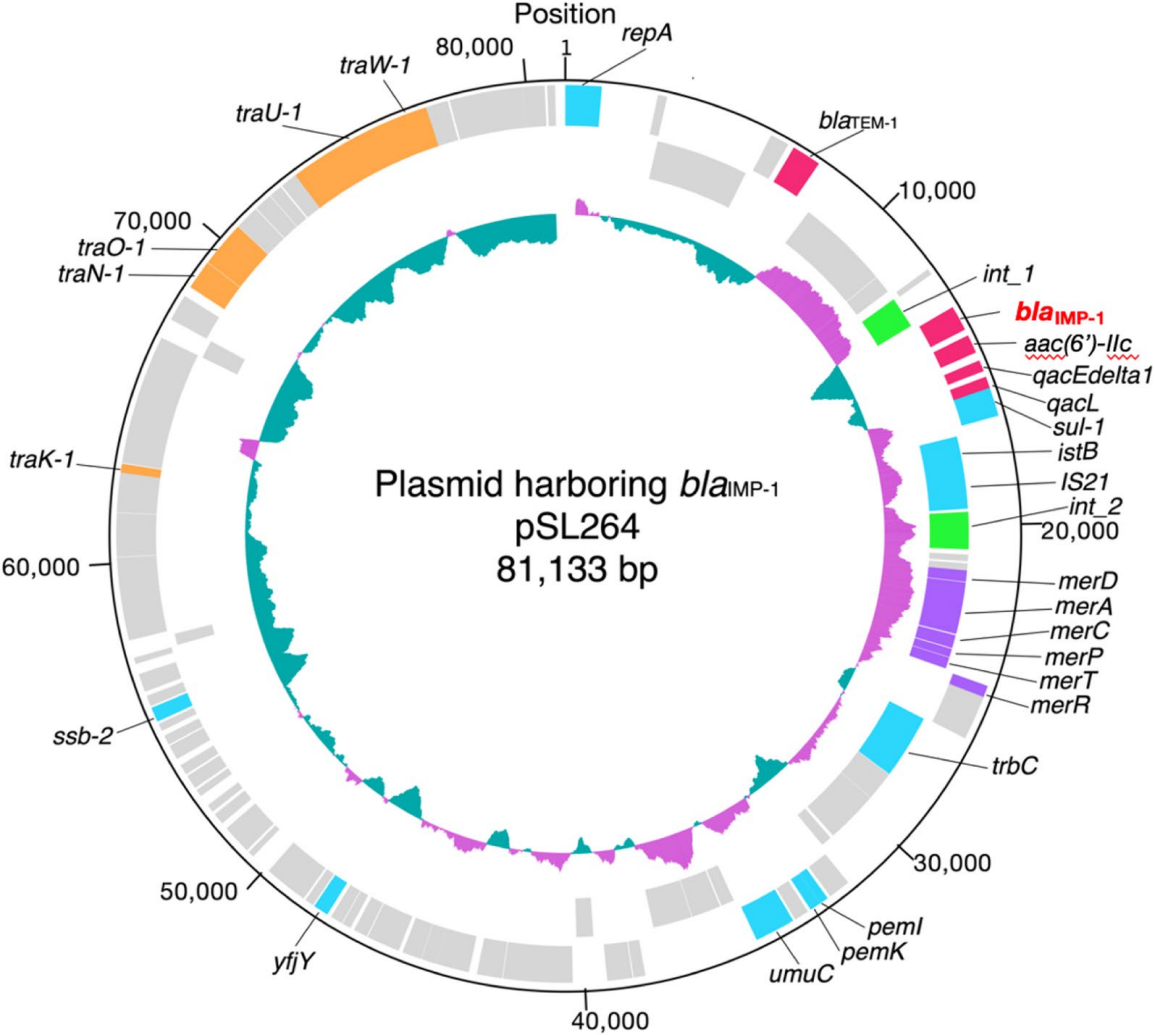
Structure of the plasmid, pSL264, harboring *bla*_IMP-1_

**Fig. 2:**
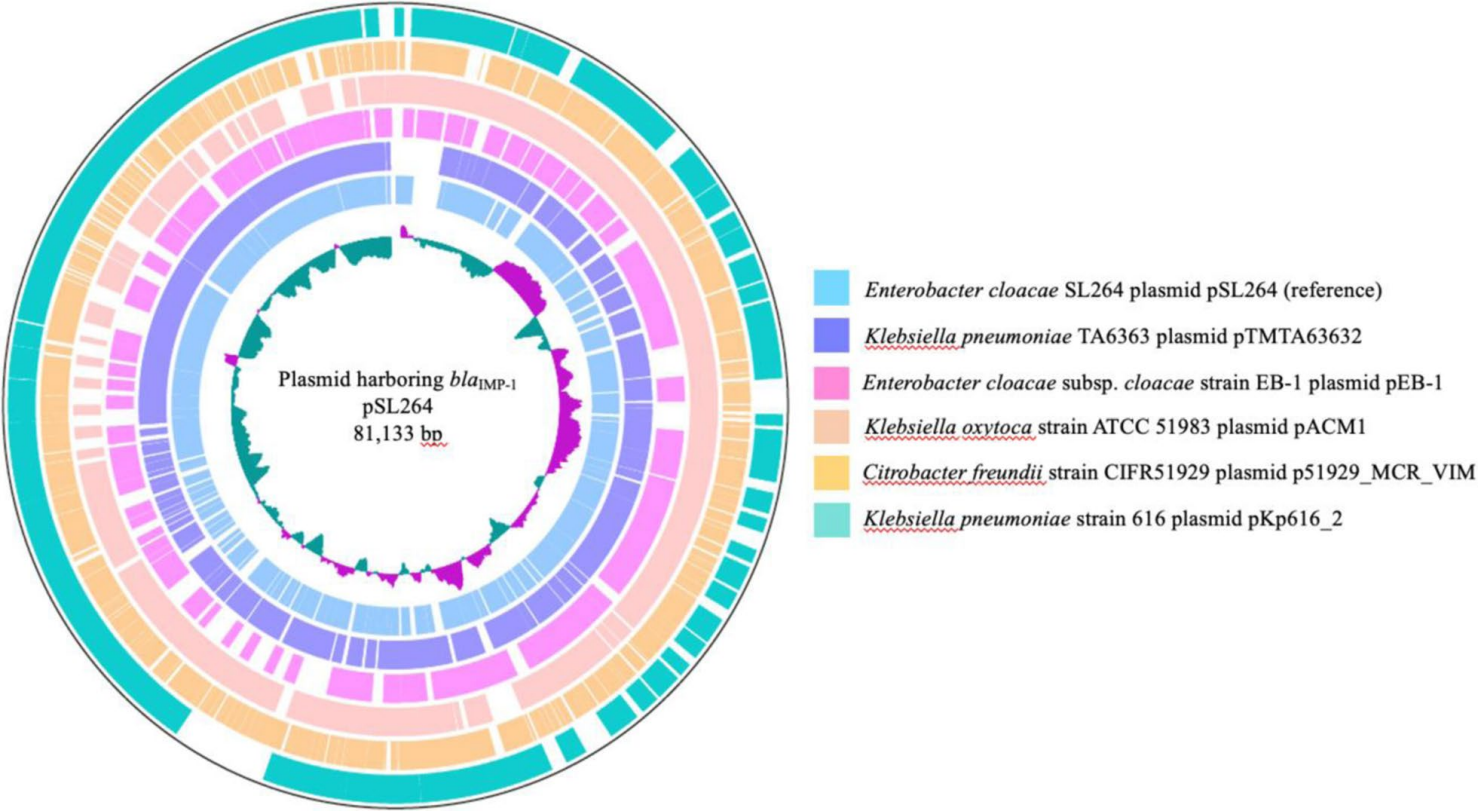
Comparative analysis of plasmids highly similar to pSL264. pSL264 was 98.51% identical to the plasmid pEB-1 in *E. cloacae* (KX230795), 98.51% identical to pACM1 in *Klebsiella oxytoca* (KJ541681), 98.51% identical to p51929_MCR_VIM in *Citrobacter freundii* CP059429), and 98.52% identical to pKp616_2 in *K. pneumoniae* (CP026497)

**Fig. 3:**
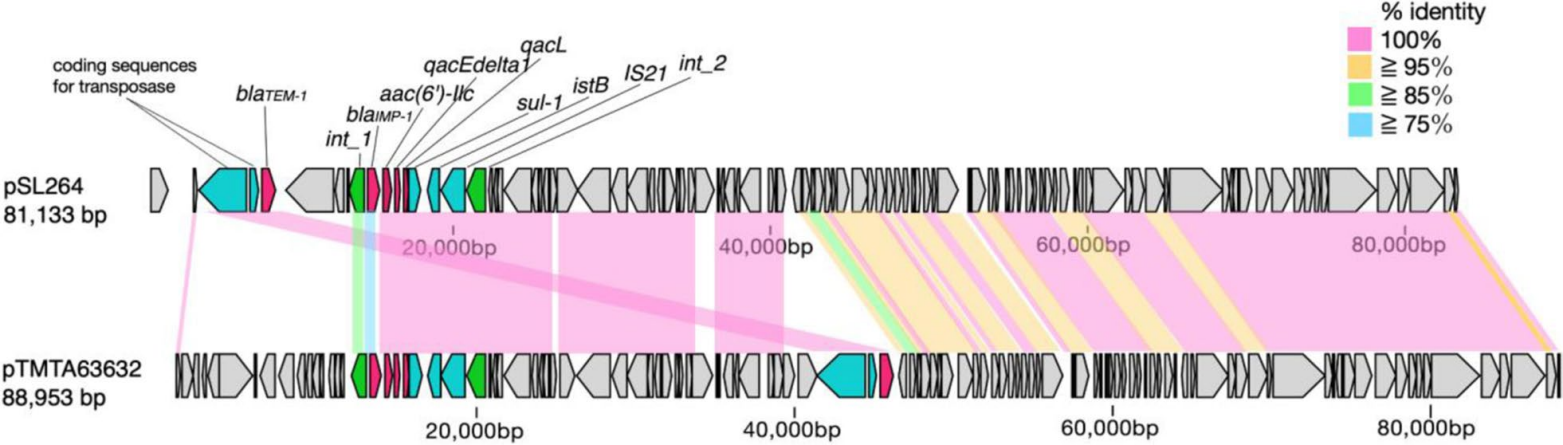
Comparative analysis of the genomes of pSL264 and pTMTA63632. The sequence *qacEdelta1-sul1-istB-*IS*21* of pSL264 was 99.25% identical to that of the plasmid TMTA63632 harboring *bla*_IMP-68_ (Accession Number AP019667) in a *K. pneumoniae* isolate obtained from a patient in Japan.c

The plasmid identified in *E. cloacae* SL264 was designated pSL264; it was found to be 81,133 bp in length and have a genetic structure of *int1-bla*_IMP-1_*-aac(6’)-IIc-qacL-qacEdelta1-sul1-istB-*IS*21* (Fig. 1). The *bla*_IMP-1_ gene was on a class 1 integron with a unique structure. The sequence of *qacEdelta1-sul1-istB-*IS*21* was 99.25% identical to that of the plasmid TMTA63632 harboring *bla*_IMP-68_ (accession number AP019667) in a *K. pneumoniae* isolate obtained from a patient in Japan (Figs. 2 and 3) [23]. The plasmid incompatibility complex of pSL264 was IncL/M. Comparative analysis showed that the plasmids in *E. cloacae* SL 264 (pSL264), *S. marcescens* SL267 (pSL267) and *K. aerogenes* SL269 (pSL269) were completely identical. This plasmid, designated pSL264, was similar to other, previously identified plasmids, including pEB-1 in *E. cloacae* with 98.51% identity (KX230795) [24], pACM1 in *Klebsiella oxytoca* with 98.51% identity (KJ541681) [25], p51929_MCR_VIM in *Citrobacter freundii* with 98.51% identity (CP059429) [26], and pKp616_2 in *K. pneumoniae* with 98.52% identity (CP026497) [27]. However, the genetic environment surrounding *bla*_IMP-1_ in pSL264 was found to be unique.

### Transferability of plasmid pSL264 harboring *bla*_IMP-1_

**Fig. 4:**
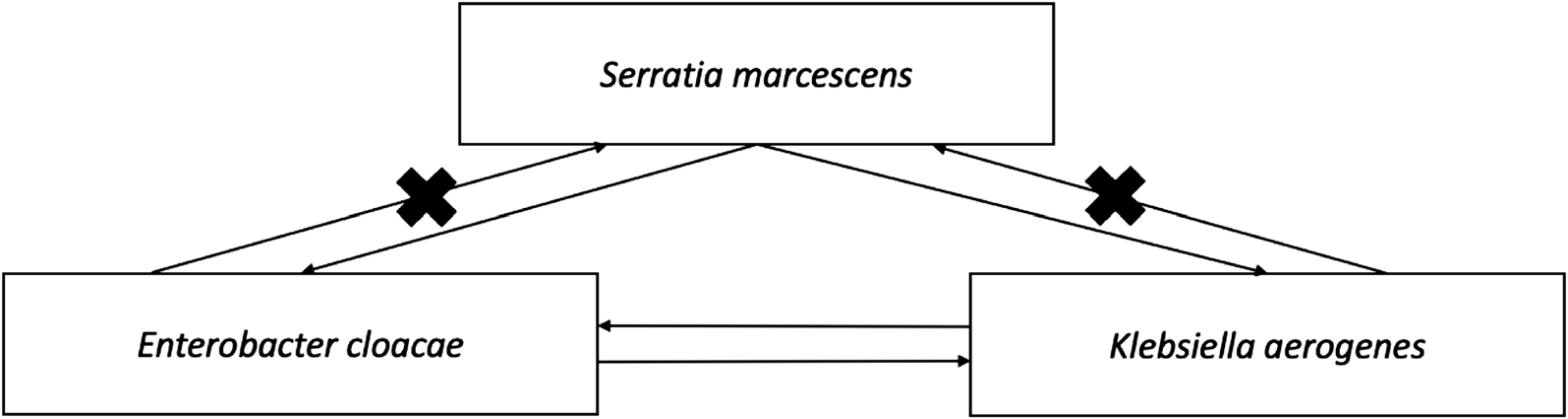
The scheme of transferability of pSL264 among *E. cloacae*, *K. aerogenes* and *S. marcescens*

Conjugation experiments showed that pSL264 could transfer from *S. marcescens* to *E. cloacae* and *K. aerogenes*, from *E. cloacae* to *K. aerogenes* and from *K. aerogenes* to *E. cloacae*. In contrast, pSL264 could not transfer from *E. cloacae* or *K. aerogenes* to *S. marcescens* (Fig. 4 and Table 2).

**Table 2:** Transferability of pSL264 among the three species of *Enterobacteriaceae*

From	To	Experiment No.		
		1	2	3
*E. cloacae*	*S. marcescens*	0	0	0
*E. cloacae*	*K. aerogenes*	0	1.3 × 10^−7^	3.7 × 10^−8^
*S. marcescens*	*E. cloacae*	7.6 × 10^−7^	5.6 × 10^−7^	1.2 × 10^−8^
*S. marcescens*	*K. aerogenes*	0	5.3 × 10^−8^	1.3 × 10^−9^
*K. aerogenes*	*E. cloacae*	3.8 × 10^−8^	5.3 × 10^−6^	2.5 × 10^−8^
*K. aerogenes*	*S. marcescens*	0	0	0

## Discussion

This study indicated that the plasmid, pSL264, transferred among three species of bacteria, *E. cloacae*, *K. aerogenes* and *S. marcescens,* in a single patient. However, our conjugation experiments indicated that pSL264 did not equally transfer among these species. Rather, it transferred from *S. marcescens* to *E. cloacae* and *K. aerogenes*, but not from *E. cloacae* or *K. aerogenes* to *S. marcescen*, suggesting that, in this patient, pSL264 was initially present in *S. marcescens* and subsequently spread to the other species. Although transferability rate in vitro was not that high, treatment with carbapenem for more than 28 days may have triggered the transfer of resistance.

This study had several limitations. First, this was a single center study. The transferability of pSL264 may not be general, and external validation may be required. Second, although in vitro conjugation experiments suggested that *S. marcescens* initially harbored pSL264, *E. cloacae* was isolated from this patient earlier than the other two species.

IncL/M plasmids harboring *bla*_IMPs_ and *bla*_NDMs_ in *Enterobacteriaceae* may be emerging and spreading, especially in Asian countries. An IncL/M plasmid was first reported in a multidrug-resistant strain of *Morganella morganii* isolated in South Africa in 1972 [28]. These plasmids are now commonly identified among environmental and clinical isolates [29, 30]. Because these plasmids are carriers of genes encoding carbapenemases, mostly OXA-48 carbapenemases, they can be regarded as a public health threat [23, 28, 30–44].

To our knowledge, this is the first report of an IncL/M plasmid harboring *bla*_IMP-1_ in three species of *Enterobacteriaceae* isolated from an individual patient*.* This plasmid may have spread clonally in the hospital environment, suggesting the need to assess the presence of *Enterobacteriaceae* harboring *bla*_IMP-1_ in medical settings in Japan.

## Conclusion

This study found that pSL264 harboring *bla*_IMP-1_ could easily transfer among species of *Enterobacteriaceae* in a patient during long-term antimicrobial treatment. Molecular and genomic analyses of plasmids may reveal the horizontal transmission of plasmids in CPE.

## Data Availability

The chromosome and two plasmid sequences of *E. cloacae* SL264 have been deposited at GenBank under accession number AP024913, AP024914 and AP024915, respectively. The chromosome and the plasmid sequences of *S. marcescens* SL267 were AP024916 and AP024916, respectively. The chromosome and two plasmid sequences of *K. aerogenes* SL269 have been deposited at GenBank under Accession Number AP024918, AP024919 and AP024920, respectively.
